# ﻿Karyotype and reproductive traits of the unique symbiotic mealybug *Orbuspedummachinator* G.-Z. (Homoptera, Coccinea)

**DOI:** 10.3897/compcytogen.17.116550

**Published:** 2023-12-18

**Authors:** Ilya A. Gavrilov-Zimin

**Affiliations:** 1 Zoological Institute, Russian Academy of Sciences, Universitetskaya nab. 1, St. Petersburg, 199034, Russia Zoological Institute, Russian Academy of Sciences St. Petersburg Russia

**Keywords:** Chromosomes, cytogenetics, Lecanoid genetic system, ovoviviparity, scale insects

## Abstract

The karyotype and reproductive features of *Orbuspedummachinator* Gavrilov-Zimin, 2017 (Pseudococcidae) were studied for the first time. Diploid chromosome number is 18 in females. Reproduction is probably bisexual, as indicated by the presence of characteristic Lecanoid heterochromatinization of the paternal set of chromosomes in embryonic cells of about 50% of the embryos studied. The female reproductive system has a pair of lateral oviducts merged into enlarged common oviduct; the spermatheca and accessory glands are connected to the common oviduct in its proximal part. Complete ovoviviparity occurs in ontogenesis.

Females of the peculiar legless mealybug *Orbuspedummachinator* Gavrilov-Zimin, 2017 from the monotypic genus *Orbuspedum* Gavrilov-Zimin, 2017 live inside conical domiciles constructed of densely packed fungal hyphae of the sooty mold *Capnodium* sp. mixed with wax secreted by the mealybug (Fig. 1a–c). The domicile grows together with the insect, which irrigates the hyphae with honeydew. This unique animal/fungus mutualistic symbiosis was described by me in details earlier (Gavrilov-Zimin 2017) from tropical rainforests of the Malay Peninsula (southern Thailand). Such mutualistic symbiosis has never been reported for any other scale insect or for any other animals known to the author. In November 2023, I was able to revisit the type locality of *O.machinator* and collect gravid females for cytogenetic and reproductive studies. The karyotype of the species includes 18 chromosomes, quite similar in length (Fig. 1d). Such diploid number has not been previously reported for any member of the informal group “legless mealybugs”, including at least 26 nominal genera in the world fauna (Gavrilov-Zimin 2017); the other studied species have 2n = 10, 12, 16, 20, 22+ Bs, 24, 24 + Bs, or 30 (Nur et al. 1987; Gavrilov-Zimin 2016, 2020).

**Figure 1.**
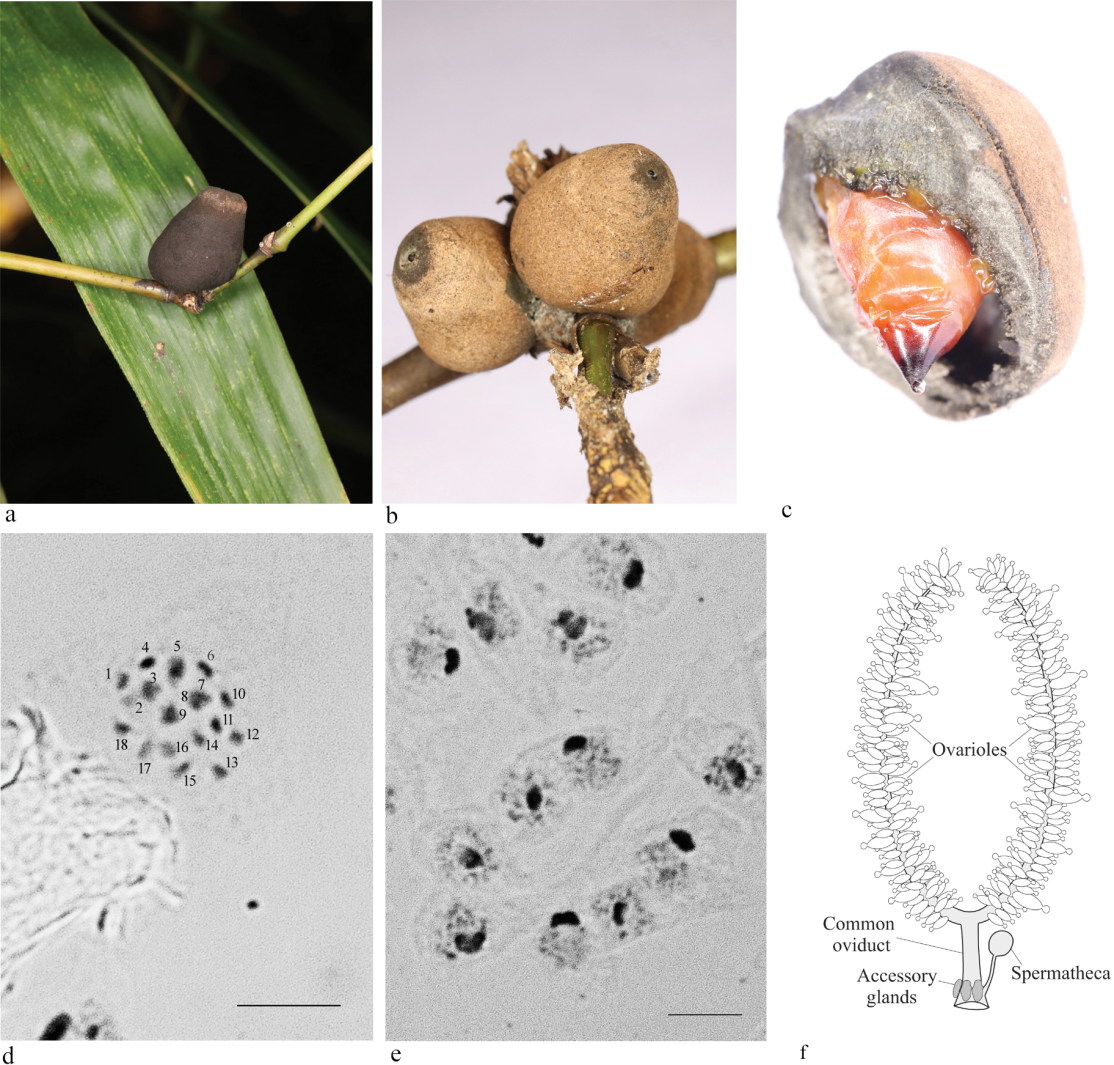
*Orbuspedummachinator*, Thailand, Khao-Sok **a** mature adult female inside a fungal domicile on twig of bamboo **b** younger females in three domiciles, **c** adult female inside a broken domicile (**a–c** photos by A.S. Kurochkin) **d** metaphase chromosomal plate in a cell of the female embryo, 2n = 18 **e** male embryo cells with heterochromatinized paternal chromosomes (deeply stained bodies) **f** scheme of the female reproductive system. Scale bar: 10 µm.

About 50% of the embryos studied contained cells with characteristic Lecanoid heterochromatinization (Fig. 1e) of the paternal chromosomes set (see Nur 1980; Gavrilov-Zimin et al. 2015 for more details). Usually in the Lecanoid system, the heterochromatic chromosome set exists in all stages of the male ontogenesis. In male meiosis, the chromosomes do not pair and separate equationally during the first division. Then, in the second division, two metaphase plates are formed, and the heterochromatic and euchromatic chromosomes segregate to the opposite poles. As a result of meiosis, quadrinucleate spermatids are formed, but only the nuclei of maternal origin produce sperm (Hughes-Schrader 1948; Nur 1980; Gavrilov-Zimin et al. 2015). Such heterochromatinization in *O.machinator* obviously indicates bisexual reproduction in the studied population. However, adult males or male larvae have not been found. This discrepancy can be explained by the probable separate life of minute males and larger females in different parts of the host plant (or even on different plants), which is a common feature of scale insects (Borchsenius 1963). Anatomical studies of the available females showed that their reproductive system is similar to that of other legless mealybugs studied (Gavrilov-Zimin 2020) and includes a pair of lateral oviducts merging into an enlarged common oviduct; the spermatheca and accessory glands are connected to the common oviduct in its proximal part (Fig. 1f). All embryonic development occurs within the ovarioles and oviducts (complete ovoviviparity). The hatched primolarvae leave the maternal fungal domicile through the apical orifice.

## References

[B1] Borchsenius NS (1963) Practical guide to the determination of scale insects of cultivated plants and forest trees of the USSR. Leningrad, 311 pp. [In Russian]

[B2] Gavrilov-Zimin IA Stekolshchikov AV Gautam DC (2015) General trends of chromosomal evolution in Aphidococca (Insecta, Homoptera, Aphidinea + Coccinea). Comparative Cytogenetics 9(3): 335–422. 10.3897/CompCytogen.v9i3.4930 26312130 PMC4547034

[B3] Gavrilov-Zimin IA (2016) Cytogenetic and taxonomic studies of some legless mealybugs (Homoptera: Coccinea: Pseudococcidae). Comparative Cytogenetics 10(4): 587–601. 10.3897/compcytogen.v10i4.10503 28123680 PMC5240511

[B4] Gavrilov-Zimin IA (2017) A remarkable example of symbiosis between an animal and a fungus in a new species of legless mealybug (Insecta: Pseudococcidae). Journal of Natural History 51(37–38): 2211–2224. 10.1080/00222933.2017.1365180

[B5] Gavrilov-Zimin IA (2020) Chromosomal and reproductive features of some Oriental and Australasian scale insects (Homoptera, Coccinea). Comparative Cytogenetics 14(3): 339–352. 10.3897/CompCytogen.v14i3.53367 32774782 PMC7387363

[B6] Hughes-Schrader S (1948) Cytology of coccids (Coccoidea-Homoptera). Advances in Genetics 2: 127–203. 10.1016/S0065-2660(08)60468-X 18103373

[B7] Nur U (1980) Evolution of unusual chromosome systems in scale insects (Coccoidea: Homoptera). In: Blackman RL Hewitt GM & Ashburner M (Eds) Insect Cytogenetics.London, 97–117.

[B8] Nur U Brown SW Beardsley JW (1987) Evolution of chromosome number in mealybugs (Pseudococcidae: Homoptera). Genetica 74: 53–60. 10.1007/BF00055094

